# Microbial metabolites and the gut-lung axis in non-small cell lung cancer immunotherapy: evidence boundaries and translational implications

**DOI:** 10.3389/fmed.2026.1890287

**Published:** 2026-08-04

**Authors:** Fangxiong Zheng, Yuchen Zhang, Zhongxin Lu

**Affiliations:** 1 Department of Medical Laboratory, The Central Hospital of Wuhan, Tongji Medical College, Huazhong University of Science and Technology, Wuhan, China; 2 School of Laboratory Medicine, Hubei University of Chinese Medicine, Wuhan, China

**Keywords:** gut microbiota, gut-lung axis, immune checkpoint inhibitors, immune-related adverse events, microbial metabolites, non-small cell lung cancer, precision medicine

## Abstract

Immune checkpoint inhibitors (ICIs) have changed the treatment of non-small cell lung cancer (NSCLC), but response, acquired resistance and immune-related adverse events (irAEs) remain variable. Gut microbiome features and microbial metabolites may contribute to this variation through systemic immunity, gut-lung communication and the tumor immune microenvironment. This structured narrative review synthesizes direct and near-direct NSCLC or lung-cancer immunotherapy evidence, including cohorts ranging from 37 to 556 patients, a phase III chemoimmunotherapy ancillary cohort with 270 baseline fecal samples and recent TOPOSCORE-based community-ecology studies. Clinical evidence reports recurrent but inconsistent associations among microbial diversity, *Akkermansia*-related states, community topology, functional microbial features, survival, treatment-duration questions and toxicity, with substantial variation by region, treatment setting and endpoint. Mechanistic studies support plausible pathways involving short-chain fatty acids (SCFAs), bile acids, tryptophan-related metabolites, inosine and exploratory bacterial extracellular vesicle-associated signals. Few studies, however, connect microbiome, metabolite, immune and clinical outcome data within the same patients. We use an evidence-boundary framework to state what each evidence layer can support. In the current evidence base, microbiome signals are better suited to prospective validation, trial design and communication of uncertainty than to routine microbiome testing or intervention outside regulated studies.

## Introduction

The central challenge in NSCLC immunotherapy is no longer whether antitumor immunity can be activated, but why only a subset of patients achieve durable benefit. PD-L1 expression, tumor mutational burden, oncogenic driver status, treatment line and regimen choice account for part of this heterogeneity, but they do not fully explain primary resistance, acquired resistance or immune-related adverse events (irAEs) (1–6). As immune checkpoint inhibitors (ICIs) are increasingly used in first-line, combination, dual-immunotherapy and perioperative settings, host-level factors, including infection, exposure to antibiotics and proton pump inhibitors (PPIs), diet, nutritional status, mucosal barrier integrity and systemic metabolism, have become increasingly important for clinical interpretation.

The gut microbiome represents one such host ecological layer. It may shape ICI response and toxicity through microbial metabolites, microbe-associated molecular patterns, mucosal immune regulation, and systemic inflammation, with downstream effects on lung immune homeostasis and the tumor immune microenvironment (7–10). However, biological plausibility should not be equated with readiness for routine clinical implementation. The clinical value of any microbiome signal depends on population directness, sampling temporality, platform reproducibility, confounding control, external validation, and interventional safety.

A further challenge is that different microbial compartments are often discussed together despite carrying different evidentiary meanings. Gut microbiome data primarily capture intestinal and systemic ecological input; lung microbiome data reflect local mucosal conditions; and tumor-associated microbiome data raise additional issues of low biomass, contamination control and spatial interpretation. These compartments may be connected, but they are not interchangeable. A gut-lung axis claim is strongest when microbial composition, microbial functional output, peripheral immune phenotypes, tumor immune features and clinical endpoints align within the same patient cohort (11–18).

This review asks what level of claim current evidence can support in NSCLC immunotherapy. We examine whether gut microbiome features are associated with ICI response, survival and toxicity; how microbial metabolites and gut-lung communication may connect intestinal ecology with systemic and tumor immunity; and which microbiome-based strategies justify validation studies, remain mechanistic hypotheses or should not be presented as clinical recommendations. The evidence-boundary framework is used to clarify these distinctions after the evidence has been synthesized.

## Evidence sources and interpretation approach

### Search and source traceability

We used a structured narrative approach with database-assisted source identification and source-level claim-role traceability. The main search was finalized on 17 May 2026, followed by targeted pre-submission surveillance on 24 May 2026 and reviewer-directed surveillance through 30 June 2026. Supplementary Table 1 lists the search blocks, source-identification routes, source-role fields, aggregate non-retention categories and reviewer-directed additions. Counts are provided for source traceability rather than formal screening accounting because this article was not designed as a systematic review, meta-analysis or formal risk-of-bias assessment.

We prioritized sources that addressed ICI-treated NSCLC, lung-cancer immunotherapy cohorts, gut-lung axis biology, microbial metabolites, irAEs, microbiome-relevant exposures such as antibiotics and PPIs, diet, probiotics, FMT, defined microbial interventions or clinical interpretation limits for microbiome-guided precision medicine. Direct NSCLC and near-direct lung-cancer evidence was synthesized before claim-use assignment. Pan-cancer, melanoma and preclinical studies were used as contextual or mechanistic evidence rather than as direct NSCLC validation.

Study quality and claim strength were judged qualitatively by population directness, sample size, sampling time, confounding control, platform reproducibility, functional validation, external validation, incremental utility and readiness for routine clinical use. Box 1 defines the claim-use categories used after the study-level evidence has been presented.

BOX 1 **Evidence-boundary rules for microbiome claims.**

Candidate association. Interpretation: In an NSCLC cohort receiving ICIs, a microbiome- or metabolite-related feature is associated with ORR, PFS, OS, or irAEs. What is missing: paired metabolomics, immune or tumor-microenvironment (TME) data, or external validation. Clinical implication: useful for hypothesis generation, not routine testing or treatment selection.

Association supported by mechanistic evidence. What it means: at least two biological layers align, such as microbiome plus metabolite or metabolite plus immune/TME evidence. What is missing: the layers may come from different cohorts, tumor types, or experimental systems. Clinical implication: strengthens plausibility but does not establish NSCLC-specific causality.

Future stratification-study hypothesis. What it means: a signal has enough longitudinal and clinical structure to justify validation in future cohorts. What is missing: calibration, external validation or incremental value beyond standard variables. Clinical implication: supports study design, not treatment assignment.

Trial rationale. What it means: biological plausibility is supported by early ecological, feasibility or safety data. What is missing: randomized clinical benefit and safety closure. Clinical implication: supports protocol-defined trials only.

**FIGURE 1:**
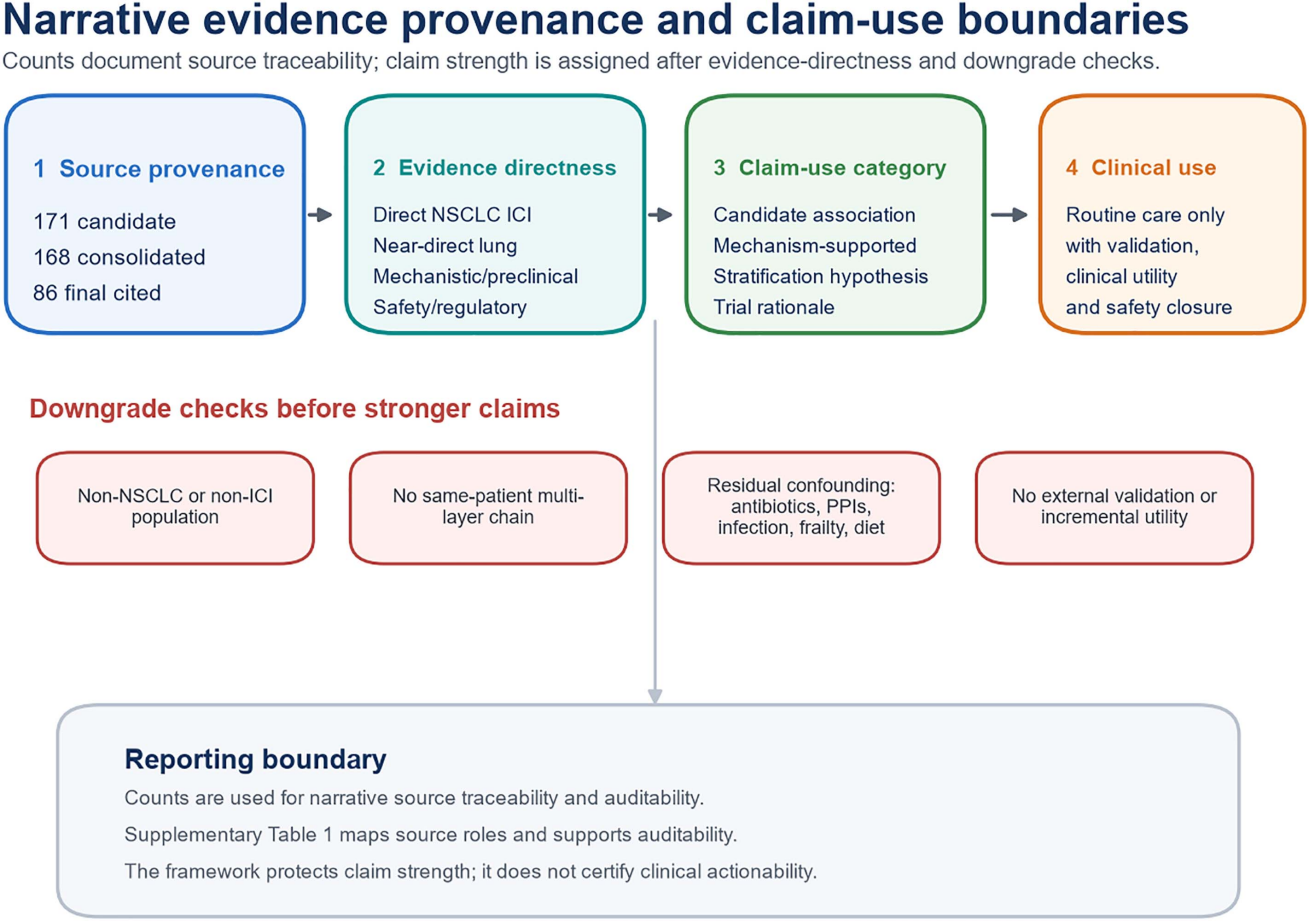
Narrative evidence-provenance and claim-use boundary schematic. The figure summarizes source-identification paths, evidence-directness tiers, claim-use assignment and downgrade triggers used in this structured narrative review. Counts support narrative traceability; Supplementary Table 1 provides the detailed source-role fields.

Not ready for routine care. What it means: validation, safety support, incremental predictive value or protocol-defined intervention evidence is insufficient. Clinical implication: this category blocks routine microbiome testing, treatment selection, commercial probiotic use and non-trial FMT. Figure 1 gives a compact audit trail for source construction, evidence-directness tiers, claim-use assignment and downgrade triggers; Supplementary Table 1 provides the detailed source-role fields.

## Evidence synthesis and interpretation limits

### Direct NSCLC evidence

Table 1 reports each study by cohort type and sample size, population and treatment setting, sampling platform, analysis performed, clinical endpoint, key quantitative finding and interpretation boundary; the narrative below follows the same order so that cohort type, analytical approach and endpoint linkage are stated consistently. The most direct cohorts remain heterogeneous in all three respects. Jin et al. studied a direct NSCLC cohort of 37 Chinese patients on nivolumab, enrolled from CheckMate 078 and CheckMate 870, using serial-stool 16S rRNA sequencing with parallel peripheral immune profiling, and correlated higher baseline diversity with RECIST response and prolonged PFS (19). Hakozaki et al. studied a prospective 70-patient Japanese cohort on single-agent anti-PD-1/PD-L1 therapy, using pretreatment 16S rRNA (V3-V4) sequencing, and correlated diversity and antibiotic exposure with ORR, PFS, OS and irAEs (20). Song/Zhang et al. studied a 63-patient Chinese cohort on PD-1 inhibitors, using paired metagenomic and functional-metabolite profiling, and correlated microbial structure and function with PFS grouping (21). Derosa et al. studied a 338-patient advanced-NSCLC cohort on PD-1 blockade, using shotgun metagenomics with tumor-transcriptomic and mouse experimental subsets, and correlated *Akkermansia muciniphila* with ORR and OS in models adjusted for PD-L1, antibiotics and performance status (22). Liu et al. studied a real-world, mixed-cancer cohort of 150 patients treated with anti-PD-1 inhibitors, including 102 patients with NSCLC, rather than an NSCLC-only cohort. Ninety patients (60%) developed irAEs; 65 had grade 1–2 events and 25 had grade 3–5 events. The five-microbial model comprised Streptococcus, Paecalibacterium, Stenotrophomonas, Faecalibacterium and unidentified Lachnospiraceae; Streptococcus, Paecalibacterium and Stenotrophomonas were more abundant in severe irAEs, whereas Faecalibacterium and unidentified Lachnospiraceae were more abundant in mild irAEs. The model distinguished patients without irAEs from those with severe irAEs with an AUC of 0.66 (23). Du et al. studied a 7-patient single-arm exploratory cohort receiving FMT plus ICI rechallenge after prior immunotherapy, using stool, metabolome and T-cell-receptor exploratory analyses, and reported safety, objective response, disease control and survival outcomes (24). Hakozaki/Tanaka et al. studied the 270-sample baseline ancillary biomarker cohort of the phase III JCOG2007 (NIPPON) first-line chemoimmunotherapy trial in treatment-naive advanced NSCLC, using 16S rDNA sequencing, and correlated microbial structure and selected genera with OS and serious adverse events (25). Three further studies extend this evidence from single cohorts to community-ecology scoring and treatment-duration questions: a TOPOSCORE development-and-validation study built from 245 NSCLC samples and validated in additional NSCLC and genitourinary cohorts (26), a strain-level analysis of 556 NSCLC patients resolving the Akkermansia signal at the SGB9226 level (27) and a retrospective 24-month ICI-discontinuation cohort applying stool TOPOSCORE after the 24-month landmark (28).

**TABLE 1:** Quantitative study-level anchors for representative NSCLC or lung-cancer ICI studies directly relevant to gut microbiota, TOPOSCORE, treatment duration and immunotherapy. The table emphasizes sample size, treatment setting, endpoint family, quantitative findings, validation status and interpretation limits before assigning claim-use categories.

Representative study	Population and treatment setting	Sample size and cohort type/design anchor	Sampling, platform and analyses performed	Clinical endpoint(s) linked to microbial features	Key quantitative or study-level finding	Interpretation boundary
Jin et al. (19)	Chinese advanced NSCLC receiving nivolumab anti-PD-1 therapy	37 patients enrolled from CheckMate 078 and CheckMate 870; direct NSCLC cohort	Serial stool at start, clinical evaluation and progression; 16S rRNA sequencing; diversity/stability analysis with parallel peripheral immune profiling	RECIST response and PFS	Responders had higher baseline diversity and more stable microbiota; high-diversity patients had significantly prolonged PFS. Responder-enriched taxa included *Alistipes putredinis*, *Bifidobacterium longum* and *Prevotella copri*; high diversity aligned with memory CD8 + T-cell and NK-cell signatures.	Small Chinese cohort; no externally validated diversity threshold or current-regimen decision rule.
Hakozaki et al. (20)	Japanese advanced NSCLC receiving single-agent anti-PD-1/PD-L1 therapy	70 patients; prospective baseline fecal-sample cohort	Pretreatment stool; 16S rRNA V3-V4 sequencing; diversity, antibiotic-exposure and taxonomic association analyses	ORR, PFS, OS and irAEs	Reported ORR was 34%, median PFS 5.2 months and median OS 16.2 months. Baseline antibiotics were linked with lower alpha diversity and underrepresentation of Clostridiales, Ruminococcaceae UCG-13 and *Agathobacter*; lower diversity was observed in poorer OS strata among antibiotic-free patients.	Response and toxicity need separate models; organ-specific irAE prediction and external validation remain unresolved.
Song/Zhang et al. (21)	Chinese advanced NSCLC receiving PD-1 inhibitor therapy	63 patients; direct NSCLC cohort with microbial and metabolite layers	Pretreatment stool; metagenomic and functional/metabolite analysis; microbial-structure and PFS-group comparison using 373.5 G sequencing data	PFS grouping and microbial function	Median PFS was 7.0 months and median OS was not reached. The PFS ≥ 6-month group had higher baseline beta-diversity and enrichment of *Parabacteroides* and *Methanobrevibacter*, whereas *Veillonella*, Selenomonadales and Negativicutes were enriched in PFS < 6-month patients.	Moves beyond taxa to function, but requires immune/TME closure and independent validation.
Derosa et al. (22)	Advanced NSCLC receiving PD-1 blockade	338 patients; direct NSCLC cohort with modeling and experimental subsets	Pretreatment stool; shotgun metagenomics; response/survival modeling adjusted for PD-L1, antibiotics and performance status; tumor-transcriptomic and mouse subsets	ORR, OS, PD-L1 and antibiotic-adjusted outcomes	*Akkermansia muciniphila* was associated with ORR and OS in models including PD-L1, antibiotics and performance status; > 4.8% *Akkermansia* with selected *Clostridium* features was linked with resistance.	Candidate ecological signal; not a universal single-taxon marker without strain/function validation, calibration and external validation.
Derosa et al. (26)	NSCLC, renal-cell and urothelial cancer patients receiving cancer immunotherapy	245 NSCLC samples used to build networks; validation in 254 additional NSCLC patients and 216 genitourinary cancer patients	Shotgun metagenomics; species-level co-abundance network analysis; TOPOSCORE construction/validation; 21-bacterial qPCR probe translation	ICI outcome, survival-risk stratification and prospective assay feasibility	Identified 37 resistance-associated metagenomic species (SIG1) and 45 response-associated species (SIG2). TOPOSCORE combined SIG topology with *Akkermansia* quantification and was externally validated across cohorts and tumor types.	Strong method-level advance and future stratification-study hypothesis; still needs prospective clinical utility and decision-impact testing.
Birebent et al. (27)	Advanced cancer cohorts with a large NSCLC component receiving chemo-immunotherapy or immunotherapy	955 patients across 4 cohorts; NSCLC *n* = 556 with 141 longitudinal paired NSCLC specimens	Stool shotgun metagenomics; MetaPhlAn4 species profiling; strain-level Akk9226 quantification; multivariable survival, longitudinal phenotype-stability and intestinal-dysfunction marker analyses	OS, PFS, Akk9226 phenotype stability and barrier-dysfunction surrogates	Akk9226 absence was linked with worse prognosis in univariable NSCLC analysis (HR 0.78, 95% CI 0.61–0.98; *p* = 0.033). Akk9226 High > 4.799% occurred in 48/556 (9%) and was associated with reduced OS versus Akk9226 Low in multivariable analysis (HR 1.79, 95% CI 1.18–2.73; *p* = 0.007).	Complicates binary *Akkermansia* framing; over-dominance may mark dysbiosis/barrier dysfunction rather than beneficial colonization.
Liu et al. (23)	Real-world anti-PD-1-treated mixed-cancer cohort; 102/150 patients had NSCLC	150 patients; mixed-cancer toxicity cohort with an NSCLC subset	Baseline stool; 16S rRNA V3-V4 sequencing; model based on Streptococcus, Paecalibacterium, Stenotrophomonas, Faecalibacterium and unidentified Lachnospiraceae	irAE grade	90/150 patients (60%) developed irAEs; 65 had grade 1–2 events and 25 had grade 3–5 events. The 5-microbial model distinguished no-irAE from severe-irAE patients with AUC 0.66.	Toxicity association only; aggregate irAEs, cancer-type heterogeneity and timing limit NSCLC-specific inference.
Du et al. (24)	Advanced NSCLC after prior immunotherapy, receiving FMT plus ICI rechallenge	7 patients, all male; median age 55 years; 5/7 had ≥ 3 prior therapy lines	Oral healthy-donor FMT capsules plus camrelizumab rechallenge; stool microbiome, metabolome and T-cell-receptor exploratory analyses	Safety, ORR, DCR, PFS and OS	No grade ≥ 4 AEs were observed; only grade 1 FMT-related AEs were reported. One patient achieved PR and one achieved SD, with PFS 14.6 and 8.1 months; median PFS was 1.5 months and OS 12.1 months.	Supports regulated trials only; no randomized control and incomplete safety/benefit closure for routine use.
Hakozaki/Tanaka et al. (25)	Treatment-naive advanced NSCLC in phase III JCOG2007 (NIPPON) chemoimmunotherapy	270 baseline fecal samples from 295 trial participants; modern first-line ancillary biomarker cohort	Baseline fecal samples; 16S rDNA sequencing; survival, serious-adverse-event and exploratory treatment-arm association analyses	OS, serious AEs and exploratory regimen-specific associations	Microbial structures differed by OS > 12 and > 18 months. *Fusicatenibacter*, *Butyricicoccus* and *Blautia* were enriched in longer-OS strata. In the NIC arm, *Fusicatenibacter* and *Butyricicoccus* were linked with better OS (HR 0.56 and 0.52), whereas Prevotellaceae NK3B31 was linked with higher mortality risk (HR 2.33).	Modern-regimen association, not a routine microbiome test or treatment-selection rule without independent validation and incremental utility testing.
Bonato et al. (28)	Advanced NSCLC patients who completed 24 months of ICI without progression	123 treated ≥ 18 months; 35 completed 24 months; 31 eligible for analysis	24-month stool WGS TOPOSCORE with FDG-PET and ctDNA where available; landmark OS, PFS and PFS24 analyses after 24 months	OS, PFS and PFS24 after the 24-month landmark	Among 31 eligible patients, 68% continued ICI and 32% discontinued therapy. After median follow-up of 59.1 months, OS did not differ by continuation versus discontinuation (*p* = 0.9012). Favorable microbiota showed a non-significant PFS24 trend versus dysbiosis (81% vs 44%; *p* = 0.0870).	Hypothesis-generating de-escalation context; small retrospective cohort, not a standalone treatment-duration rule.

Treatment context is a major boundary. Many microbiome-ICI observations come from advanced or later-line anti-PD-1/PD-L1 settings, whereas modern first-line chemoimmunotherapy data are still emerging. The JCOG2007 ancillary study supports regimen-specific microbiome analysis in treatment-naive advanced NSCLC, but first-line chemoimmunotherapy-associated signals still need independent validation, calibration and incremental utility testing before they can inform treatment choice. Signals from single-agent immunotherapy, chemoimmunotherapy, dual-checkpoint blockade, dual ICI plus chemotherapy, neoadjuvant or perioperative regimens, rechallenge settings and long-responder de-escalation settings should not be treated as interchangeable. Chemotherapy, corticosteroid exposure, antibiotics, infection, nutrition, surgery, tumor burden kinetics and endpoints such as pathologic response, event-free survival, disease-free survival or PFS after a 24-month landmark may all change both microbiome signals and clinical meaning (3–6, 19–28).

*Akkermansia muciniphila* is an informative but context-dependent ecological lead, and its relationship with immunotherapy outcome is non-monotonic rather than simply ‘more is better’. In the Derosa et al. cohort of advanced NSCLC treated with PD-1 blockade, intestinal *A. muciniphila* was associated with objective response and overall survival in models adjusted for PD-L1, antibiotics and performance status, yet a relative abundance above roughly 4.8% together with selected *Clostridium* features was instead linked with resistance (22). Derosa et al. were the first to describe this three-state (‘trichotomous’) pattern in the Nature Medicine cohort: both absence and overabundance were unfavorable, whereas an intermediate, physiological abundance was more favorable. Birebent et al. subsequently confirmed and refined the pattern at strain level in 556 NSCLC patients: absence of *A. muciniphila* SGB9226 was associated with worse prognosis in univariable analysis (HR 0.78, 95% CI 0.61–0.98; *p* = 0.033), whereas a high relative abundance above 4.799% occurred in 48 of 556 patients (9%) and was associated with reduced OS in multivariable analysis (HR 1.79, 95% CI 1.18–2.73; *p* = 0.007) (27). Species-level detection still does not establish strain-level function, functional equivalence across regions or incremental utility beyond established clinical variables.

Derosa et al. moved the field beyond single taxa by developing TOPOSCORE in Cell in 2024. Metagenomic analysis of 245 NSCLC fecal samples was used to build species-level co-abundance networks, identifying 37 metagenomic species associated with resistance (SIG1) and 45 associated with response (SIG2). TOPOSCORE combined these species-interacting group signals with Akkermansia quantification and was validated in 254 additional NSCLC patients and 216 genitourinary cancer patients. The score was translated into a 21-bacterial qPCR probe set and assessed prospectively in NSCLC and other tumor settings. This work shifts interpretation from isolated taxa to ecological topology, but routine use still requires prospective decision-utility testing (26).

Bonato et al. extended microbiome profiling from initial response prediction to the question of ICI duration after 2 years. In a retrospective Gustave Roussy cohort, 123 patients had received at least 18 months of ICI, 35 completed 24 months and 31 were eligible after exclusion of patients with progression exactly at 24 months. Twenty-one patients continued ICI and 10 discontinued by physician decision. After a median follow-up of 59.1 months, OS did not differ between continuation and discontinuation. TOPOSCORE-defined favorable gut composition showed a non-significant trend toward higher PFS24 after the 24-month landmark than dysbiotic composition. The result is hypothesis-generating rather than a treatment-stopping rule, but it identifies a testable role for microbiome profiling in future de-escalation algorithms (28).

Routy et al. linked the gut microbiome, including *Akkermansia muciniphila*, with PD-1-based immunotherapy efficacy across epithelial tumors and used responder-derived FMT transfer to germ-free mice to support biological plausibility (29). Gopalakrishnan et al. and Matson et al. showed that melanoma anti-PD-1 response was associated with distinct commensal configurations, immune features and experimental transferability (30, 31). Spencer et al. linked dietary fiber and probiotic exposure with melanoma immunotherapy outcomes, and Baruch et al. and Davar et al. provided proof-of-concept that FMT can promote response in selected anti-PD-1-refractory melanoma patients (32–34). Together, these studies provide biological and methodological context and support mechanistic and interventional hypotheses, but differences in tumor type, treatment history, donor selection and safety context prevent direct transfer of clinical rules into NSCLC.

Toxicity evidence indicates that microbiome research should not focus solely on improving response. Liu et al. reported that the intestinal microbiome was associated with irAEs in patients treated with anti-PD-1 inhibitors (23). Studies of ICI-associated colitis and systemic toxicity further implicate mucosal barrier function, local inflammation, regulatory T cells, Th17 responses and microbial metabolites in toxicity development (35–37). However, toxicity studies need sharper temporality and phenotype definition. Colitis, pneumonitis, hepatitis, endocrine irAEs, dermatologic irAEs and systemic irAEs should not be collapsed into a single microbiome-toxicity phenotype. Pretreatment microbiome features, on-treatment microbiome shifts, post-irAE microbiome changes and changes caused by corticosteroids, immunosuppressants, antibiotics or colitis treatment are different evidence types. Without this separation, microbial differences may represent a cause, consequence or treatment effect.

Exposure to antibiotics and PPIs is one of the most common sources of misinterpretation. Antibiotics can disrupt microbial diversity and metabolite output, but patients receiving antibiotics often have infection, hospitalization, poor performance status or higher disease burden, each of which can influence survival (38–43). PPIs, corticosteroids, diet, comorbidities, ACE inhibitors and treatment line may create similar confounding or medication-context effects (44). Time-dependent exposure, protopathic bias and reverse causation are especially important: medication may precede poor outcome, but it may also mark early symptoms, infection, frailty or treatment complications. The defensible clinical implication is not that indicated antibiotics or PPIs should be stopped before ICI therapy. It is that antimicrobial stewardship, guideline-concordant prescribing and careful exposure recording should be embedded into immunotherapy studies, including exposure window, indication, duration, infection status, hospitalization and concurrent treatments.

### Counter-signals and non-replication

Counter-signals and non-replication define the boundary of microbiome claims. They should be interpreted as context and study-design signals rather than as simple failures of reproducibility. Diversity metrics are a useful example: higher diversity has been linked with response in some NSCLC cohorts, but alpha- and beta-diversity vary with geography, diet, sequencing platform, baseline inflammation and treatment regimen. These measures therefore support cohort-level hypotheses, not fixed clinical thresholds.

The same caution applies to taxa and exposure variables. Akkermansia-related findings should be read in trichotomous rather than binary terms, because both absence and over-dominance may mark unfavorable states in NSCLC (22, 27). Associations involving antibiotics and PPIs should also be treated as confounded clinical signals unless analyses account for infection, frailty, hospitalization, nutritional status, corticosteroid exposure, disease burden and performance status (38–44). This framing separates reproducible biological leads from premature clinical rules.

The same caution applies to mechanistic and interventional signals. SCFA-related mechanisms are context dependent, because concentration, tissue compartment, receptor context, the Treg/CD8 balance and toxicity phenotype all change their interpretation. FMT evidence supports a regulated trial rationale, particularly in selected rechallenge settings, but not routine pre-treatment FMT or commercial adjunctive use. Aggregate irAE signals should not be treated as organ-specific predictors of colitis, pneumonitis, hepatitis, endocrine, dermatologic or systemic toxicity without phenotype-specific sampling and timing. Across all of these examples the underlying problem is the same: a statistically detectable association is not, by itself, a stable and transferable clinical rule.

### Microbial metabolites as functional mediators

Microbial composition is more interpretable when linked to functional output. A clinically meaningful chain would connect a gut microbial community or candidate taxon with microbial functional capacity, stool and circulating metabolite profiles, peripheral immune tone, tumor immune microenvironment features, ICI response or irAE phenotype, and independent validation beyond PD-L1, driver genes, ECOG performance status, antibiotics and treatment line (45–50). Most studies close only part of this chain. Table 2 separates metabolite classes by experimental system, compartment ambiguity, directionality and upgrade requirements rather than treating all mechanistic studies as equivalent.

**TABLE 2:** Functional mediator layers linking microbial outputs with immune steps and clinical interpretation. Rows separate measured or inferred compartment, likely source ambiguity, directionality and upgrade requirements because fecal abundance, circulating metabolites and tumor-local concentrations are not interchangeable.

Functional layer	Representative molecule or structure	Possible immune step	Evidence type	Compartment/ source ambiguity	Directionality and upgrade requirement	Clinical interpretation
Fermentation metabolites	Short-chain fatty acids (SCFAs), especially acetate, propionate and butyrate	Barrier integrity, T-cell metabolism, Treg/CD8 balance, epigenetic regulation	Smith et al. showed SCFA-dependent colonic Treg homeostasis in mice; Furusawa et al. showed butyrate-induced colonic Treg differentiation *in vitro* and *in vivo* (51, 52)	Stool, plasma and tumor-local concentrations are not interchangeable; diet and host metabolism contribute	Bidirectional/context dependent; upgrade requires same-patient stool and plasma metabolomics plus immune/TME endpoints	Plausible mediator class, not validated NSCLC clinical mediator
Bile acid metabolism	Microbial bile-acid metabolites reported in immunology models; no NSCLC-validated species	TH17/Treg balance with indirect relevance to antitumor immunity	Hang et al. showed that microbial bile-acid metabolites regulate TH17 and Treg differentiation in mouse and cellular systems (53)	Host hepatobiliary metabolism, diet, liver involvement, antibiotics and concomitant drugs can dominate signal attribution	Unresolved; upgrade requires oncology cohort data with paired stool/plasma metabolomics, immune readouts and clinical endpoints	Indirect oncology-relevant mechanism with low NSCLC readiness; not a clinical biomarker or intervention target
Purine and tryptophan-linked pathways	Inosine, with indole and kynurenine-related pathways as broader metabolic context	A2A receptor signaling, aryl hydrocarbon receptor, T-cell function and inflammatory balance	Mager et al. showed microbiome-derived inosine improved checkpoint-inhibitor efficacy in four mouse cancer models under co-stimulation conditions (54)	Microbial, host immune and tumor metabolic sources can overlap	Context dependent; upgrade requires compartment-specific metabolomics and endpoint-linked immune profiling	Inosine is mechanism-supported but preclinical and co-stimulation dependent; not clinically actionable in NSCLC
Microbial immune-priming structures	Microbial-associated molecular patterns, exploratory bacterial extracellular vesicle-associated signals and commensal immune-priming signals	Dendritic-cell maturation, antigen presentation, innate immune activation	Mechanistic experiments and early translational work (15, 48, 57, 58)	Low-biomass and host-response effects can confound source attribution	Dose and timing dependent; upgrade requires NSCLC patient validation and immune/TME linkage	Hypothesis-generating mechanism candidate
Gut-lung integration	Mucosal barrier, inflammatory mediators and immune-cell trafficking	Lung immune homeostasis and tumor immune microenvironment	NSCLC reviews, lung microbiome studies and pan-cancer mechanisms (11, 12, 17)	Stool, lung and tumor microbial compartments require separate interpretation	Unresolved; upgrade requires synchronized stool, blood, immune and tumor sampling	Organ-axis framework, not proof that stool signal equals tumor-local mechanism

Short-chain fatty acids (SCFA) evidence is biologically strong but clinically indirect for NSCLC immunotherapy. Smith et al. showed in mice that microbial SCFAs regulate the size and function of the colonic regulatory T-cell pool and protect against colitis in an Ffar2-dependent manner (51). Furusawa et al. showed that butyrate induces colonic regulatory T-cell differentiation *in vitro* and *in vivo*, improves colitis in a Rag1-/- transfer model and increases histone H3 acetylation at the Foxp3 locus (52). These studies support immune plausibility, but they do not show that fecal SCFA abundance predicts ICI response or toxicity in NSCLC. Concentration, anatomical compartment, receptor context and the balance between antitumor CD8 + immunity and regulatory T-cell activity remain critical boundaries.

Bile-acid evidence is retained only as a narrow, oncology-relevant candidate pathway. Hang et al. showed that microbial bile-acid metabolites can regulate TH17/Treg differentiation in mouse and cellular systems (53). This immune axis is relevant to checkpoint-inhibitor biology, but it is not direct evidence for NSCLC immunotherapy outcomes. In oncology patients, bile-acid profiles are also shaped by hepatobiliary function, diet, antibiotics, liver involvement and concomitant drugs. No NSCLC study has yet linked a defined bile-acid species with ICI response, survival or toxicity using paired microbiome-metabolite and clinical endpoint data. Bile acids are therefore presented as a low-readiness pathway that may inform future metabolomics analyses, not as a validated biomarker or intervention target.

Inosine is included because it offers the most mechanistically explicit link between a microbial metabolite and checkpoint-inhibitor activity, which is precisely why its relevance - and its limits - must be stated carefully. Inosine is a microbial purine metabolite that can enter the systemic circulation when treatment increases gut-barrier permeability and then acts on the adenosine A2A receptor (A2AR) on T cells. This is counterintuitive, because the adenosine-A2AR axis is classically immunosuppressive in the tumor microenvironment; the central finding of Mager et al. is that microbiome-derived inosine - from *Bifidobacterium pseudolongum*, *Lactobacillus johnsonii* and *Olsenella* species - instead enhanced checkpoint-inhibitor efficacy across four mouse cancer models, but only when T cells received concurrent co-stimulation such as that supplied by checkpoint blockade (54). Inosine is therefore relevant here as a conditional, co-stimulation-dependent enhancer of the same T-cell response that ICIs unblock, not as a free-standing immunostimulant. This co-stimulation dependence is also the principal caveat: the effect is context-specific and preclinical, so inosine should be read as mechanism-supported plausibility, not as evidence that inosine supplementation or a specific bacterial product is clinically actionable in NSCLC.

Metabolomics shifts microbiome research from taxonomic composition toward functional output. Song et al. incorporated intestinal flora structure and metabolite profiling into an NSCLC immunotherapy study, reporting a median PFS of 7.0 months, median OS not reached, 373.5 G of sequencing data, higher baseline beta-diversity in the PFS ≥ 6-month group, enrichment of *Parabacteroides* and *Methanobrevibacter* in longer-PFS patients, and functional differences across KO, COG and CAZy profiles (21). Fecal metabolite studies in lung cancer provide additional leads (55). However, fecal metabolites do not necessarily reflect plasma or tumor-local concentrations, and circulating metabolites may originate from host metabolism, drugs, or diet. Stronger evidence would come from same-patient studies that jointly measure the stool microbiome, stool and plasma metabolites, peripheral immune phenotypes, the tumor immune microenvironment, and clinical endpoints. Because the same metabolite may arise from host metabolism, diet, drug exposure, tumor metabolism, or microbial metabolism, fecal abundance alone cannot establish causal direction.

The gut-lung axis helps organize these observations at the organ-system level. Gut microbiota may influence lung immune homeostasis through metabolites, inflammatory mediators, mucosal barrier function and immune-cell migration, whereas lung inflammation and tumor immune conditions may in turn reflect systemic immune tone (11, 12, 17). Microbe-associated molecular patterns and commensal immune-priming signals can shape dendritic-cell activation, antigen presentation and antitumor immunity in experimental models (15, 48, 56–58). In NSCLC, the strongest formulation is not that gut microbiota alone explains treatment response, but that microbial functional output may shape host immune readiness and influence multiple steps of the cancer-immunity cycle.

**FIGURE 2:**
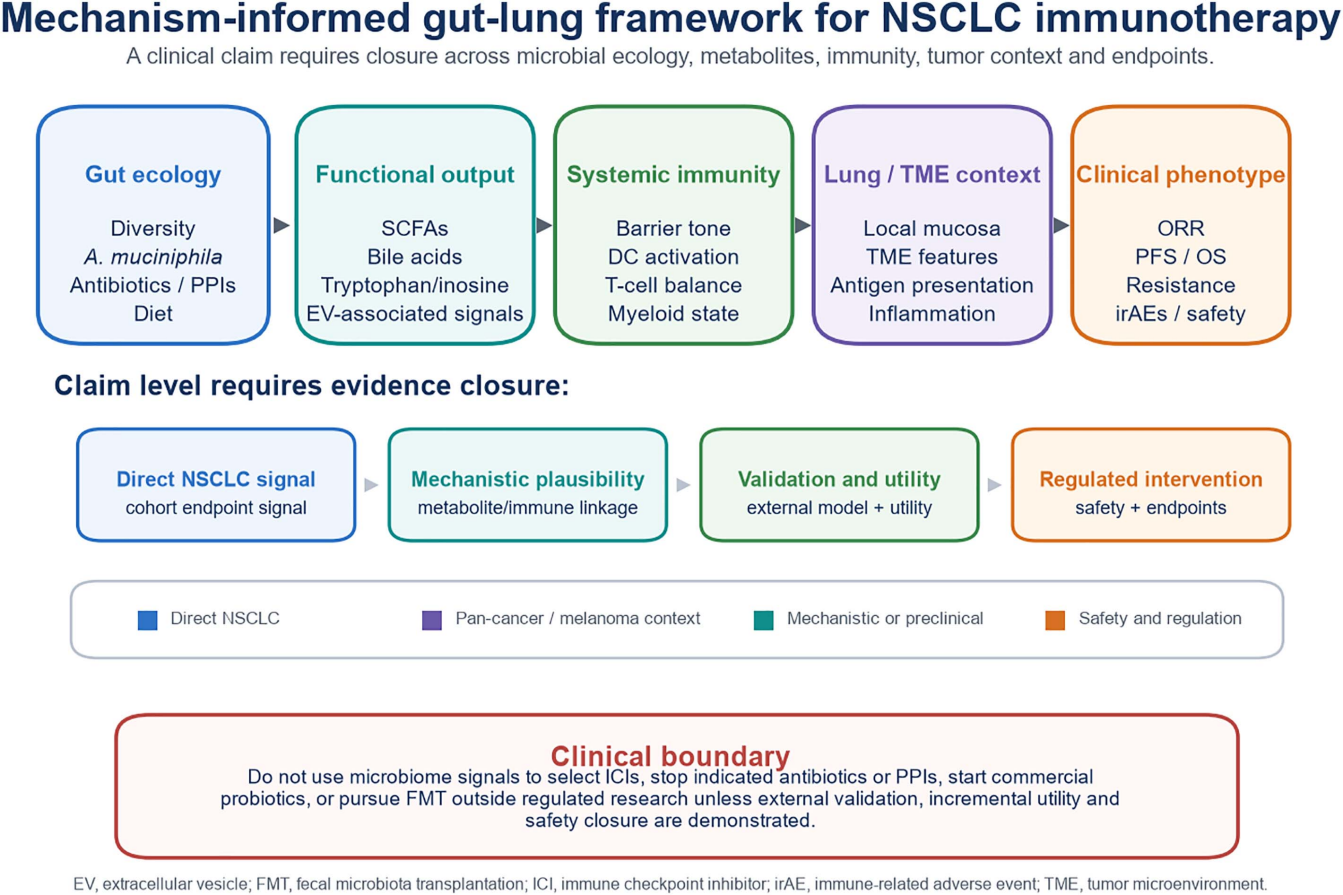
Mechanism-informed framework linking gut microbial metabolites and the gut-lung axis to NSCLC immunotherapy response and toxicity. The model separates direct NSCLC evidence from pan-cancer, melanoma and preclinical evidence. It treats the microbiome-metabolite-immune-TME pathway as a testable mechanistic chain rather than an established causal pathway. Bacterial taxa are formatted according to standard taxonomic nomenclature.

Figure 2 presents the mechanistic chain and separates direct NSCLC evidence, pan-cancer and melanoma context, preclinical mechanisms and hypothesis-generating links, because these evidence layers carry different levels of translational confidence.

### Sources of heterogeneity and reproducibility limits

Many microbiome findings in NSCLC immunotherapy are difficult to compare because study design varies across multiple levels. Direct NSCLC cohorts in the current evidence base are weighted toward Chinese and Japanese studies, with important French and broader European contributions in Akkermansia and TOPOSCORE work (19–28). This geographic structure is informative but limits universal taxon-specific interpretation because diet, host genetics, smoking background, lifestyle, medication access, sequencing platform, sample handling and local microbial ecology differ across regions. Population differences also include histology, stage, treatment line, ECOG performance status, driver mutation status, PD-L1 expression and prior treatments (59–62). Without careful adjustment and external validation across regions, a microbial signal may reflect host background, diet, healthcare context or treatment selection rather than immunotherapy biology.

Sampling time also affects interpretation. Pretreatment stool reflects the baseline ecological state but does not demonstrate stability during therapy. Early on-treatment samples may capture dynamic changes, but they are also affected by treatment, infection, diet and concomitant medication. Post-progression samples may reflect tumor burden, declining performance status and subsequent treatment rather than causality (43, 61). Longitudinal sampling before therapy, during early treatment, at first response assessment, during toxicity and at progression would better distinguish cause, accompaniment and consequence.

Detection platforms and analytical pipelines also shape microbiome-derived conclusions. 16S rRNA sequencing, shotgun metagenomics, TOPOSCORE or other ecological scoring methods, reference databases, species-level annotation, functional inference, batch correction, and statistical modeling can all influence which taxa or species-interacting groups are identified as candidates (26, 61, 63). When studies report only relative abundance, without absolute quantification, pathway-level functional data, metabolite profiling, or external validation, cross-platform robustness is difficult to assess. Future assays should, at minimum, report sampling time, sample handling and storage, DNA extraction methods, sequencing or metabolomics platforms, bioinformatics pipelines, batch-correction procedures, relative and absolute abundance strategies where available, metabolomics quality control, and cross-platform validation. For low-abundance taxa, strain-level functions, ecological topology and metabolic pathway inference, metagenomics coupled with metabolomics is more mechanistically informative than 16S analysis alone.

Clinical endpoints should also be kept distinct. Objective response rate (ORR), disease control rate (DCR), progression-free survival (PFS), overall survival (OS), treatment discontinuation, and immune-related adverse events (irAEs) capture different clinical dimensions. Single-agent immunotherapy, chemoimmunotherapy, dual immunotherapy, perioperative therapy, and later-line treatment also represent distinct immune contexts (3–6). Pooling these regimens and endpoints may cause microbial signals to reflect treatment background rather than stable immunotherapy biology.

**FIGURE 3:**
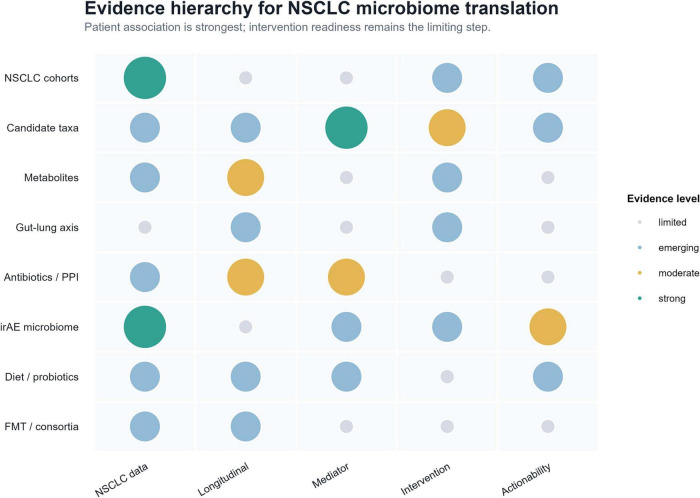
Evidence hierarchy for NSCLC microbiome interpretation. Dot size represents an ordinal qualitative summary of evidence strength across clinical directness, longitudinal design, mediator linkage, intervention evidence and actionability. It was not calculated from the number of supporting studies. Larger dots indicate stronger overall support across these dimensions. The main gap is reproducible mediator closure and validated clinical utility, not biological plausibility.

Figure 3 summarizes this evidence hierarchy. Direct NSCLC cohorts provide the most clinically proximal association evidence, but they remain limited for establishing functional mediation or interventional readiness. Mechanistic studies strengthen biological plausibility but often lack NSCLC-specific validation. Intervention studies are promising, but they remain constrained by donor selection, strain definition, dose, safety, and regulatory pathways.

### Translational feasibility and safety considerations

From a clinical safety perspective, microbiome testing, diet, probiotics, management of antibiotics and PPIs, FMT and engineered microbial strategies should remain research or stewardship questions rather than patient-level treatment instructions (64–79). These findings should not be used to alter ICI therapy, withhold indicated antibiotics or PPIs, initiate commercial probiotics or pursue FMT outside protocol-defined and regulated clinical research (64–78). At this stage, exposures should be recorded, microbiome-metabolite-immune profiles should be tested prospectively and microbiome results should not be used to select, withhold or modify NSCLC immunotherapy (64–79).

Clinicians should record microbiome-relevant exposures for trial interpretation rather than order microbiome panels to select ICIs (67, 68). If microbiome-based precision medicine is pursued, models should be validated across regions, diets, sequencing platforms, sample-processing pipelines and payment settings before clinical implementation (67, 68).

Clinical toxicity interpretation should remain anchored in established irAE management guidelines. Microbiome or metabolite findings may help generate toxicity hypotheses, but diagnosis, grading, corticosteroid or immunosuppressive treatment, treatment interruption and rechallenge decisions should follow oncology toxicity guidelines rather than microbiome test results (64–66).

**FIGURE 4:**
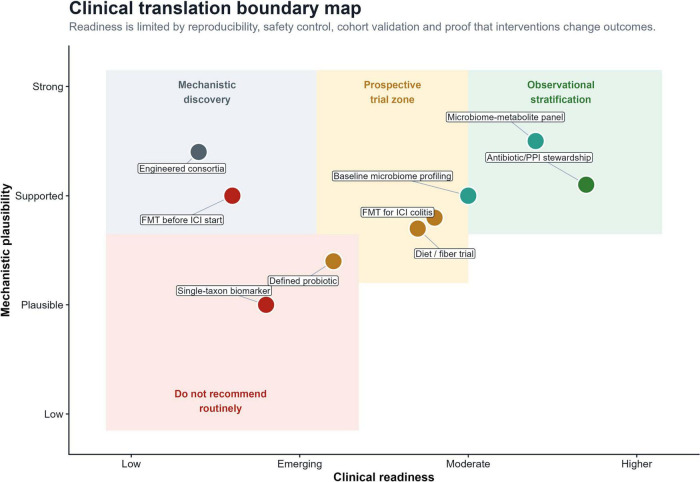
Clinical interpretation map for microbiome strategies in NSCLC immunotherapy. Placement was assigned qualitatively according to mechanistic plausibility and clinical readiness. Clinical readiness considered external validation, incremental utility beyond established clinical variables, protocol-defined intervention evidence, regulatory and safety requirements, and whether the strategy could support routine clinical use. Items in the not-routine-use zone lack sufficient external validation, decision utility or safety closure.

The near-term role of microbiome testing is to generate hypotheses for future stratification studies and mechanistic subtyping, not to guide independent clinical decisions. Pretreatment diversity, *A. muciniphila*, selected commensals and metabolite features may be evaluated as candidate benefit or toxicity signals within externally validated research models, but translational value requires calibration, external validation and incremental performance beyond clinical variables (19, 20, 22, 25, 67, 68). A clinically plausible model should integrate PD-L1, driver mutation status, histology, treatment regimen, antibiotic and PPI exposure, nutritional status, inflammatory markers, microbial features and metabolites. Microbiome variables should complement host ecological information rather than replace tumor molecular testing. Plausible research uses include enrollment enrichment, exploratory toxicity-risk modeling within prospective studies and microbiome-metabolite mechanistic subtyping, but not direct treatment selection. Table 3 summarizes near-term translational positions and conclusions that should not be drawn, and Figure 4 maps these strategies by mechanistic plausibility and clinical readiness.

**TABLE 3:** Translational strategies, required conditions and conclusions that should not be drawn. The central principle is that measurable does not mean actionable, and modifiable does not mean clinically indicated.

Translational path	Near-term position	Required conditions	Conclusion that should not be drawn	Main risk
Baseline microbiome profiling	Hypothesis generation for future stratification studies	Standardized sampling, platform consistency, calibration, external validation and incremental predictive value	A single genus can replace PD-L1, driver genes, treatment regimen or clinical staging	False positives, batch effects and geographic variation
Microbiome-metabolite panel	Mechanistic subtyping for prospective validation	Matched stool, blood metabolites, immune phenotypes and clinical endpoints	Fecal metabolites directly represent tumor-local concentrations	Source ambiguity and host metabolic confounding
Exposure to antibiotics and PPIs	Contextual exposure and stewardship variable	Clear indication, time window, duration, infection, hospitalization status and concurrent medication	Antibiotics or PPIs are absolute contraindications	Confounding by indication and disease severity
Dietary factors and probiotic exposure	Protocol-defined, strain-specific, safety-monitored research direction	Strain, dose, viable count, diet background, adherence, safety and product quality recorded	Commercial probiotics routinely enhance ICI efficacy	Variable composition, unclear mechanism, contamination risk and overmarketing
FMT and defined consortia	Regulated clinical-trial strategy, not routine adjunctive treatment	Donor screening, multidrug-resistant organism (MDRO)/pathogen control, quality standards, trial oversight, safety monitoring and long-term follow-up	FMT before ICI initiation is a routine NSCLC adjunct	Infection, MDRO transmission, batch variation, regulation and ethics
Defined consortia, postbiotics and engineered microorganisms	Investigational, mechanism-driven early translation	Clear target, dose relationship, engraftment stability and immune endpoint	Use is justified without human safety data	Ecological disturbance, off-target effects and unknown long-term safety

Exposure to antibiotics and PPIs should first be treated as contextual exposures and stewardship variables, not treatment targets unless independently indicated. Exposure records should include timing around ICI initiation, drug class, duration, indication, infection site, hospitalization and concurrent therapy. Only when baseline microbial disruption, treatment-period infection and progression-related medication are separated can antibiotic-outcome relationships be interpreted responsibly (38–41). In clinical practice, the cautious position is standard antimicrobial stewardship and guideline-concordant PPI prescribing, not categorical discontinuation of indicated medication.

Dietary factors and probiotic exposure are common and recordable, but intervention should remain protocol-defined, strain-specific and safety-monitored. Diet may influence immune background through fiber, nutritional status and microbial metabolites, but effects depend on baseline microbiota, weight change, treatment context and adherence (40–42). Probiotics are not a single therapeutic class. Commercial products, research-grade strains, postbiotics and defined consortia differ in composition, quality control and mechanism (69, 70). Studies that record only “probiotic use” without strain, dose, viable count, duration, diet background and adherence cannot establish efficacy or safety.

Commercial overinterpretation is a risk when mechanistic plausibility is mistaken for clinical evidence. Clinical practice guidance emphasizes that probiotic recommendations are indication- and strain-specific, whereas live biotherapeutic products (LBPs) require defined manufacturing and quality-control standards distinct from commercial supplement use (71, 72). For translation, commercial probiotics, defined microbial consortia, FMT-derived products and live biotherapeutic products (LBPs) should not be treated as interchangeable categories.

FMT and defined microbial consortia have greater ecological remodeling potential and higher safety requirements. FMT from responders or healthy donors has been investigated as a way to reshape recipient ecology in selected ICI-resistant, rechallenge or toxicity research settings (24, 33, 34, 68, 80). Baruch et al. and Davar et al. showed in anti-PD-1-refractory melanoma that FMT could promote clinical responses in selected trial participants, but those studies used melanoma populations, protocol-specific donors and regulated monitoring (33, 34). They support biological rationale and NSCLC trial design, not direct transfer to routine NSCLC pre-treatment FMT. References on probiotics, FMT or immune modulation outside NSCLC provide general safety and microbiome-modulation context rather than NSCLC-specific efficacy evidence (70, 73, 74). FMT therefore remains a regulated clinical-trial strategy rather than a routine pre-treatment adjunct in NSCLC.

Donor screening, pathogen transmission, multidrug-resistant organism risk, batch variation, delivery route, timing, concomitant chemotherapy, immunosuppression, irAE colitis, long-term safety and regulatory oversight all require explicit control. Clinical guidance and regulatory safety communications on fecal microbiota products emphasize donor screening, pathogen testing, traceability and oversight because transmission of multidrug-resistant or pathogenic organisms can cause serious adverse events (75–78). Currently approved fecal microbiota products are indicated for prevention of recurrent *Clostridioides difficile* infection after antibacterial treatment, not for NSCLC immunotherapy optimization (76–78).

More standardized approaches, including defined microbial consortia, postbiotics, metabolite modulation and engineered microorganisms, remain investigational. These strategies may reduce donor variability and safety uncertainty, but their mechanisms, dose-response relationships, engraftment stability, immune endpoints, off-target effects and long-term safety require prospective validation (49, 50, 55, 56, 79, 81). Until the evidence matures, the appropriate path is mechanism-driven, protocol-regulated trials rather than routine adjunctive treatment. LBP development requires defined chemistry, manufacturing and control information, including strain identity, viable dose, contamination control and assessment of antimicrobial resistance and transferability (72).

### Evidence-boundary matrix and interpretation limits

Table 1 summarizes study-level results; Table 4 identifies the missing evidence elements that prevent each microbiome or metabolite signal from supporting a stronger claim. A microbiome claim can move toward stronger interpretation only when direct NSCLC evidence, paired microbiome, metabolite, immune and outcome data from the same patients, longitudinal sampling, confounding control, external validation, incremental clinical utility and intervention safety are progressively satisfied.

**TABLE 4:** Evidence-boundary upgrade matrix.

Claim or translational use	What current evidence supports	Missing chain element	Downgrade trigger	Minimum evidence needed for upgrade
Single-taxon *Akkermansia* signal	Candidate ecological association in NSCLC cohorts; the non-monotonic (trichotomous) abundance pattern was first described by Derosa et al. and refined at strain level (Akk9226) by Birebent et al. (22, 27)	Strain/function validation, metabolite linkage and incremental utility	Absence and over-dominance can both be unfavorable; geography and platform can change thresholds	Prospective multi-region validation with strain-level profiling, calibration and clinical-utility testing
TOPOSCORE and community ecology	Future stratification-study hypothesis supported by ecological topology, external validation and qPCR translation (26, 28)	Decision-utility proof in a defined NSCLC clinical decision	Score performance may vary by treatment line, regimen, geography and assay platform	Prospective NSCLC trials showing incremental value beyond PD-L1, clinical covariates and exposure records
Microbiome-guided ICI duration	Hypothesis-generating post-24-month de-escalation signal (28)	Adequately powered prospective comparator and complete multiomic data	Small retrospective long-responder cohort; no treatment-interaction test	Prospective de-escalation algorithm with microbiome, ctDNA, imaging and safety endpoints
Metabolite-mediated mechanism	Mechanism-supported association for SCFAs, bile acids and inosine from immunology and mouse cancer models (51–54)	Same-patient microbiome-metabolite-immune/TME-outcome closure in NSCLC	Fecal abundance may not represent plasma or tumor-local exposure	Longitudinal stool/plasma metabolomics plus immune phenotyping, tumor tissue and clinical endpoints
Microbiome-toxicity prediction	Candidate association between baseline microbiome features and aggregate irAEs (23, 35–37)	Organ-specific irAE definitions, temporality and treatment-effect separation	Steroids, antibiotics, colitis treatment and infection may be consequences rather than causes	Prospective phenotype-specific irAE cohorts with pre-irAE and post-treatment sampling
FMT or microbial intervention	Trial rationale in selected rechallenge/resistance settings and melanoma proof-of-concept (24, 33, 34, 80)	Randomized NSCLC benefit, donor/strain safety and ecological endpoint closure	Melanoma or mixed-cancer response cannot be directly transferred to NSCLC	Protocol-regulated trials with donor screening, MDRO testing, immune endpoints and long-term safety follow-up
Routine clinical microbiome testing	Not ready for routine care; useful for exposure recording and validation-study design	External validation, calibration, net benefit and implementation pathway	Commercial tests may overstate actionability before utility is shown	Decision-curve or net-benefit analysis demonstrating added value beyond standard oncology variables

### Applying the evidence-boundary framework

Practical evidence-boundary workflow. Step 1 identifies source directness: direct NSCLC ICI evidence, mixed-cancer evidence, preclinical or mechanistic evidence, or intervention and safety context. Step 2 checks data-chain completeness, including microbiome, metabolite, immune/TME readout, longitudinal sampling and clinical endpoint linkage. Step 3 applies downgrade checks for confounding control, external validation, incremental utility and safety closure. Step 4 assigns the most conservative claim-use category supported by the evidence.

The evidence summarized above sets the limits of the framework. The framework does not replace study-level synthesis; it states what level of claim each evidence layer can support. We apply it only after considering population directness, sample size, endpoint definition, quantitative findings, confounding control, validation, incremental utility and safety closure.

We applied downgrade triggers when evidence was indirect for NSCLC, came from mixed-cancer or non-ICI populations, relied only on animal or *in vitro* systems, or did not include same-patient microbiome-metabolite-immune/TME linkage, longitudinal sampling, metabolomics, immune or tumor-microenvironment readouts, clinical endpoints, confounder control, external validation, incremental utility beyond standard clinical variables or intervention safety closure. Key confounders included antibiotics, PPIs, corticosteroids, diet, infection, hospitalization, performance status and treatment regimen. Biological plausibility alone did not upgrade a claim.

Three examples show how the framework changes interpretation. First, a single-taxon response signal supports a candidate ecological association, but it does not support routine microbiome testing, treatment selection or a universal predictor. Upgrading this claim would require prospective NSCLC validation, strain-level or functional profiling, paired metabolomics, immune/TME endpoints, calibration and incremental utility. Second, TOPOSCORE and the *Akkermansia* trichotomy support future stratification-study hypotheses because they add ecological topology, external validation or survival-adjusted structure, but they still require prospective decision-utility testing before clinical deployment. Third, FMT supports regulated trial rationale in selected resistance, rechallenge or toxicity-research settings, but it does not support routine pre-treatment FMT, commercial adjunctive use or toxicity prevention outside protocol-defined studies.

The framework is a clinical communication and study-design checklist rather than a numerical evidence score or a substitute for GRADE. It is intended to align microbiome claims with evidence strength, directness, same-patient multi-layer support, external validation, incremental utility and intervention safety closure.

## Discussion

Current evidence supports the gut microbiome, microbial metabolites and the gut-lung axis as biologically plausible contributors to heterogeneity in NSCLC immunotherapy. Direct and near-direct evidence should be considered before framework application. NSCLC cohorts link microbial diversity, selected taxa, functional microbial features, TOPOSCORE-defined community ecology, antibiotic exposure, serious adverse events, irAEs and treatment-duration questions to clinical outcomes, including emerging first-line chemoimmunotherapy evidence from JCOG2007 and post-24-month ICI de-escalation hypotheses (19–28). Pan-cancer and melanoma landmarks provide biological, dietary, FMT and validation context, but they should not be treated as equivalent to NSCLC-specific clinical validation (29–34, 82). Additional pan-cancer reviews discuss gut microbiota-TME interactions and tumor immunomodulation; these sources provide background support rather than direct NSCLC validation (83–85).

Detailed claim-use categories and upgrade requirements are provided in Box 1 and Table 4; the remainder of this discussion focuses on study design and clinical translation.

Future studies should move beyond taxonomic discovery toward testable, multi-layer evidence chains. Cohorts should ideally collect stool samples before and during therapy, stool and blood metabolite profiles, peripheral immune phenotypes, tumor tissue immune features and clinical outcomes from the same patients. A candidate microbe or pathway becomes more credible when it aligns with metabolite changes, immune phenotype shifts and clinical endpoints over time. If only cross-sectional abundance differences are available, conclusions should remain at the candidate-association level.

For precision medicine, routine clinical value requires more than statistical association. A microbiome-informed model should improve prediction or decision-making beyond established variables, including PD-L1 expression, driver alterations, histology, treatment regimen, inflammatory markers, exposure to antibiotics and PPIs, nutritional status and performance status. Microbiome signals may function as prognostic markers of frailty, inflammation, infection exposure, nutritional status or medication context rather than predictive markers of ICI-specific benefit unless treatment interaction and incremental utility beyond established clinical variables are demonstrated. Trial-readiness should therefore be judged against a model based only on standard clinical variables, prespecified primary endpoint, safety endpoint, ecological endpoint, external validation, decision-curve or net-benefit analysis, and subgroup calibration across regions, diets, sequencing platforms and healthcare systems.

Microbiome interventions require especially cautious interpretation because modifiability does not equal clinical indication. Diet, probiotics, postbiotics, FMT, defined microbial consortia, and engineered microorganisms differ substantially in mechanism, quality control, and risk. Melanoma studies support the biological relevance of dietary fiber, caution against unselected probiotic use, and provide proof-of-concept that FMT may overcome anti-PD-1 resistance in selected trial settings, but these data do not establish routine NSCLC intervention use (32–34). Lower-complexity strategies may first be captured through exposure recording and standard oncology nutrition or antimicrobial-stewardship practices. Higher-risk strategies, especially FMT and engineered microorganisms, require donor or strain control, pathogen screening, regulatory oversight, ecological endpoints, safety monitoring, and long-term follow-up (24, 33, 34, 68, 73, 74, 80).

The logic of this framework may be applied to other immunotherapy-treated malignancies if cancer-specific directness is reassessed. Its main strength in pan-cancer settings is the separation of cross-tumor biological plausibility from disease-specific clinical readiness. Melanoma and mixed-cancer studies strengthen the plausibility that commensal ecology, diet, probiotics, FMT and cross-cohort validation influence ICI response, but the final claim-use category should be assigned separately for each malignancy, regimen and endpoint (30–34, 82). Pan-cancer application is limited by differences in tumor biology, treatment line, ICI regimen, exposure to antibiotics and PPIs, diet, sampling compartment, toxicity phenotype and background standard-of-care therapy. The framework can support cross-cancer comparison and study design, but it cannot justify direct transfer of treatment-selection rules into NSCLC.

Several limitations should be considered. NSCLC-specific evidence spans heterogeneous cohort sizes, from small exploratory cohorts to larger datasets, and remains limited by single-center designs, cross-sectional sampling, variable sequencing platforms and heterogeneous endpoint definitions. Examples of larger datasets include Derosa et al. (*n* = 338), Birebent et al. (*n* = 556 NSCLC patients within 955 patients across four cohorts), the TOPOSCORE development and validation cohorts (245 NSCLC samples plus 254 additional NSCLC and 216 genitourinary cancer patients), and the JCOG2007 ancillary cohort (270 fecal samples from 295 patients). Direct NSCLC data are geographically uneven, with many cohorts from Chinese or Japanese populations and several influential French or European cohorts. This distribution limits broad transfer of taxon-specific thresholds because diet, host genetics, lifestyle, medication practice and microbial background differ across regions. The source set was limited to English-language literature, and negative and non-replicated microbiome findings were not systematically captured; the synthesis should therefore not be read as a prevalence estimate of all positive and negative evidence. Regional variation, diet, antibiotic exposure, PPI use, infection, hospitalization and performance status may all contribute to residual confounding. Stool-, lung- and tumor-associated microbial signals were interpreted as compartment-specific findings, and observations from one compartment were not taken as evidence of a mechanism operating in another.

Future work should follow a staged precision-medicine pathway anchored in prospective, multicenter NSCLC cohorts. A next-generation cohort should recruit across East Asian, European and other geographic settings; collect stool before therapy, during early treatment, at response assessment, during irAEs and at progression; and pair stool shotgun metagenomics with stool and plasma metabolomics, peripheral immune phenotyping, tumor tissue immune features and standardized clinical outcomes. It should capture antibiotics, PPIs, corticosteroids, diet, infection, hospitalization, nutritional status, smoking, treatment regimen, treatment line, PD-L1, driver alterations and ECOG performance status as prespecified covariates. Within this pathway, the primary short-term priority is decision-utility testing - showing whether microbiome-derived signals add incremental value beyond PD-L1, tumor genotype and other established clinical variables - supported by cross-platform external validation of ecological scores such as TOPOSCORE. More specific questions, including the *Akkermansia* trichotomy and microbiome-informed treatment-duration decisions, are worth pursuing but should be framed as secondary, proportionally weighted aims rather than headline objectives. Regulated intervention trials evaluating diet, defined microbial strains, postbiotics, FMT or engineered microorganisms should proceed only after calibration, incremental utility and safety closure, with ecological remodeling endpoints and clinical outcomes built into the trial design.

Recent studies available through 30 June 2026 clarify the current boundary of microbiome-guided NSCLC immunotherapy. The Cell TOPOSCORE study provided a validated community-ecology scoring approach built from 245 NSCLC samples and validated in additional NSCLC and genitourinary cohorts, shifting emphasis from isolated taxa toward species-interacting groups. Birebent et al. showed in 556 NSCLC patients that *Akkermansia muciniphila* SGB9226 is not a simple binary biomarker because both absence and over-dominance can mark unfavorable states. Bonato et al. applied TOPOSCORE to the novel question of ICI continuation beyond 24 months and found a hypothesis-generating PFS24 trend, not a treatment-stopping rule. The JCOG2007 (NIPPON) ancillary biomarker study analyzed 270 treatment-naive advanced NSCLC patients with baseline fecal samples in a phase III chemoimmunotherapy trial and reported associations between microbial structure, selected genera, OS, serious adverse events and possible regimen-specific effects, but these results still require external validation and incremental-utility testing before clinical use. The phase 2 FMT-LUMINate trial extended FMT plus immunotherapy evidence into first-line ICI-treated NSCLC and melanoma populations, but it remains a protocol-defined clinical-trial strategy rather than support for routine pre-treatment FMT or commercial microbiome intervention. A 2025 NSCLC chemo-immunotherapy study linking gut microbiota and SCFA-related signals to treatment response further supports regimen-specific validation of metabolite pathways, but it does not remove the need for same-patient metabolomics, immune/TME linkage, calibration, external validation and incremental clinical utility testing (25–28, 80, 86).

## Conclusion

Gut microbial metabolites and the gut-lung axis provide a biologically plausible basis for investigating heterogeneity in NSCLC immunotherapy outcomes. Current evidence mainly supports candidate associations, mechanism-informed hypotheses, future stratification studies and regulated trial rationales. It does not yet support routine microbiome testing, microbiome-guided treatment selection, commercial probiotic use or non-trial FMT. The most concrete short-term priority is prospective, multicenter validation of community-ecology and metabolite-linked biomarkers, together with decision-utility testing of whether such signals add value beyond established clinical variables for response and toxicity prediction. More specific questions, including the *Akkermansia* trichotomy and microbiome-informed treatment-duration decisions after 2 years, are of interest but are better regarded as illustrative secondary directions than as principal priorities. Clinical use should remain limited to exposure recording, validation-study design and communication of uncertainty, while ICI treatment, antibiotics, PPIs and supportive medications remain anchored in established clinical indications.
